# Male Breast Cancer Originating in an Accessory Mammary Gland in the Axilla: A Case Report

**DOI:** 10.1155/2012/286210

**Published:** 2012-12-01

**Authors:** Jun Yamamura, Norikazu Masuda, Yoshinori Kodama, Hiroyuki Yasojima, Makiko Mizutani, Keiko Kuriyama, Masayuki Mano, Shoji Nakamori, Mitsugu Sekimoto

**Affiliations:** ^1^Department of Surgery, Osaka National Hospital, 2-1-14 Hoenzaka, Chuo-ku, Osaka 540-0006, Japan; ^2^Department of Pathology, Osaka National Hospital, 2-1-14 Hoenzaka, Chuo-ku, Osaka 540-0006, Japan; ^3^Department of Radiology, Osaka National Hospital, 2-1-14 Hoenzaka, Chuo-ku, Osaka 540-0006, Japan

## Abstract

Carcinoma of an accessory mammary gland is an extremely rare tumor. A 61-year-old male patient presented with a hard mass measuring 85 mm × 51 mm in the left axilla. Incisional biopsy histopathologically showed an adenocarcinoma compatible with breast carcinoma originating in an accessory mammary gland. Systemic examinations revealed no evidence of malignant or occult primary lesion in the bilateral mammary glands or in other organs. Neoadjuvant chemotherapy was performed for the locally advanced axillary tumor and reduced the tumor to 55 mm in size, and, then, he could undergo complete resection with a negative surgical margin in combination with reconstructive surgery to fill the resulting skin defect with a local flap of the latissimus dorsi muscle. The patient has presented with no metastatic lesion in four years since the operation. This unusual case shows that neoadjuvant chemotherapy is an effective and tolerated therapy for advanced accessory breast cancer in the axilla.

## 1. Introduction

Cases of adenocarcinoma in the axilla are uncommon and can be regarded as sebaceous or sweat gland cancer, mammary carcinoma arising in an accessory mammary gland, or metastatic lymph nodes from breast cancer or another primary cancer [1–3]. Herein, we describe a rare case of a male patient with an axillary malignant tumor which could be histopathologically compatible with breast cancer arising in an accessory mammary gland.

## 2. Case Report

**Figure 1:**
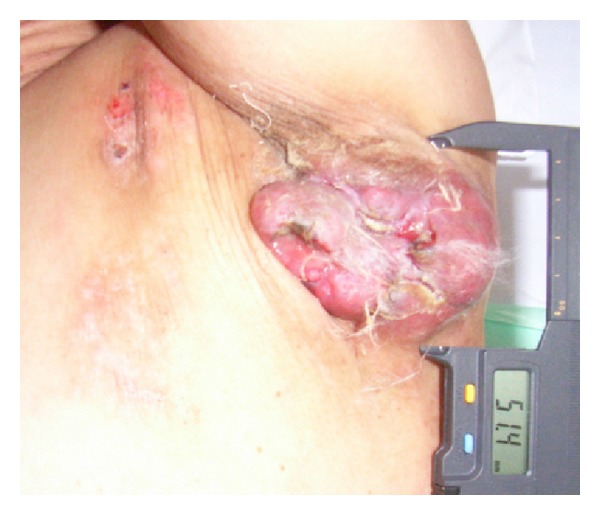
An irregular, immobile, and hard mass, measuring roughly 85 mm × 51 mm, exposed in the left axilla with slight bleeding.

**Figure 2:**
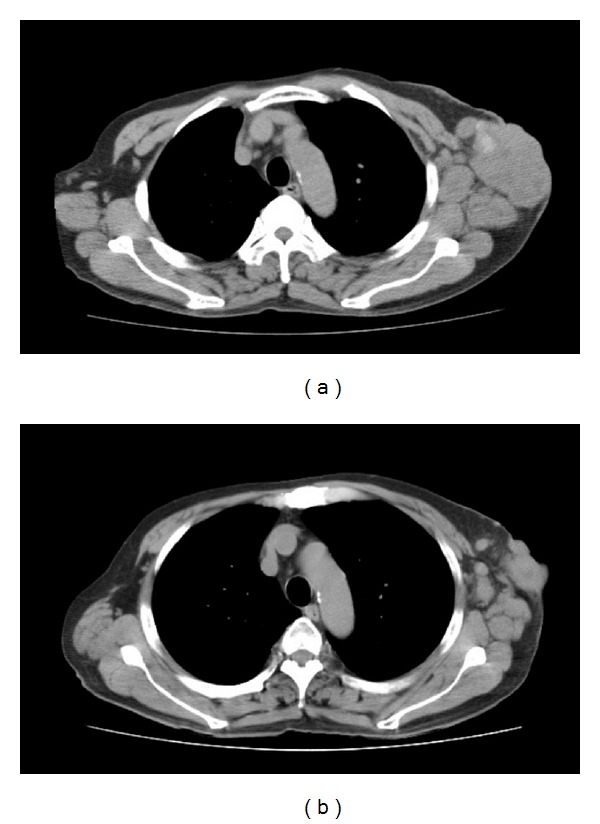
(a) Computed tomography (CT) showed an exposed and lobulated 77 mm soft tissue density mass with faint calcification in wide contact with the skin, enlarged lymph nodes in the left axilla, and small round lymph nodes less than 10 mm in the mediastinum. (b) After the neoadjuvant chemotherapy, the tumor was reduced to 51 mm in diameter, while the small mediastinum lymph nodes remained about the same in size.

A 61-year-old man first noticed a small subcutaneous nodule in the left axillary area in 2005. The nodule gradually increased in size and he was referred to our hospital in November 2007. Clinical examination revealed an irregular immobile hard mass, measuring roughly 85 mm × 51 mm in the left axilla (Figure 1). Computed tomography (CT) showed an exposed and lobulated 77 mm soft tissue density mass with faint calcification in wide contact with skin (Figure 2(a)). Also, CT showed suspicious direct involvement of the left subclavian vein, enlarged lymph nodes in the left axilla, and small round lymph nodes less than 10 mm in the mediastinum. Magnetic resonance imaging (MRI) and ultrasonography (US) revealed no primary lesion in the ipsilateral breast tail and bilateral mammary gland. Additionally, positron emission tomography (PET)/CT demonstrated no evidence of any malignant or occult primary lesions, but the axillary tumor.

**Figure 3:**
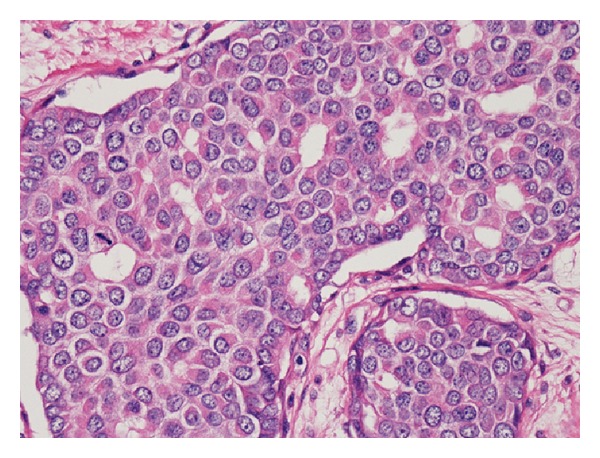
The histological examination of the incisional biopsy specimen revealed proliferation of atypical cells of variable size in the subcutis. The cells had enlarged and irregular nuclei and formed a luminal structure unconnected with the epidermis, indicating an adenocarcinoma compatible with breast carcinoma.

An incisional biopsy histologically revealed proliferation of atypical cells of variable size in the subcutis. The cells had enlarged and irregular nuclei and formed a luminal structure unconnected with the epidermis, indicating an adenocarcinoma compatible with breast carcinoma (Figure 3). Immunohistochemically, both of the estrogen receptor (ER) and progesterone receptor (PgR) were positive, and human epidermal growth factor receptor type 2 (HER2) was negative. And, the immunoexpression of proliferation factor Ki67 was high (about 24%). These findings were strongly suggestive of mammary carcinoma originating in an accessory mammary gland in the axilla. We regarded the axillary tumor as locally advanced accessory breast cancer that was difficult to resect completely and, therefore, planned neoadjuvant chemotherapy.

The patient was subjected to 4 courses of FEC chemotherapy (5-Fu at 500 mg/m^2^, epirubicine at 100 mg/m^2^, and cyclophosphamide at 500 mg/m^2^) every 3 weeks, but sequential docetaxel chemotherapy at 75 mg/m^2^ was discontinued due to a severe allergic reaction, and he, therefore, received additional 2 courses of FEC chemotherapy. The tumor was finally reduced to 55 mm in diameter, and the partial response to the neoadjuvant chemotherapy was also confirmed by CT (Figure 2(b)). Wide radical excision was then performed with preservation of the axillary vein and brachial plexus, despite suspicious involvement of the tumor. Radical axillary lymph node dissection was also performed, as well as subsequent reconstructive surgery to fill the resulting skin defect with a local flap of the latissimus dorsi muscle to preserve shoulder joint movement.

The final pathological examination of the surgical specimen showed moderately differentiated adenocarcinoma compatible with invasive ductal breast carcinoma, which was the same as the result obtained with the earlier excisional biopsy. And, the decrease of immunoexpression of Ki67 due to the effect of neoadjuvant chemotherapy was confirmed. There was no evidence of intraductal components or lymph node structures in the specimen. The surgical margin was negative. Accordingly, a course of hormone therapy (tamoxifen at 20 mg/day) was started after surgery. The patient has not presented with any metastatic lesions in the four years since the operation and has been receiving hormone therapy.

## 3. Discussion

Accessory mammary carcinoma is very rare, occurring in only 0.3%–0.6% of all cases of breast cancer and usually appears as an axillary tumor [4]. Accessory mammary tissue develops due to incomplete embryologic regression of the mammary ridge, which is composed of a portion of the galactic band that runs from the axilla to the groin [5]. Generally, accessory breast cancer must be pathologically demonstrated to be located adjacent to normal breast ducts or lobules that are not connected with the proper mammary gland, and it is also necessary to exclude the possibility of a metastatic lesion from another primary cancer [6].

In our case, systemic examinations including CT, MRI, US, and PET/CT revealed no primary malignant or occult lesions, only the axillary tumor. Although pathological examination did not show such findings as isolated normal breast tissue or intraductal spread adjacent to the main lesion in the specimen, the tumor was morphologically compatible with mammary carcinoma, and immunohistochemical study revealed ER- and PgR-positive cells. Thus, we diagnosed the axillary tumor as primary breast cancer arising in an accessory mammary gland. In any case, it is most important to plan proper therapeutic strategy for breast cancer.

In the previous literature, it has been reported that in males the incidence of accessory breast cancer is higher than that of the usual form of breast cancer [7]. In addition, at the time of diagnosis the majority of accessory breast cancer patients were in an advanced clinical stage, with nodal metastasis or unresectable tumor size [7–9]. Thus, the prognosis of accessory breast cancer may be worse than that of breast cancer arising in proper mammary gland, although no long-term follow-up data regarding the prognosis of accessory breast cancer is available [10].

In our case, the patient presented with a locally advanced axillary tumor, which could not be removed completely, and the patient, therefore, underwent neoadjuvant chemotherapy. As a result, the lesion was reduced in size after 6 courses of FEC chemotherapy, and it was clinically considered as a partial response. There have been few reports on neoadjuvant chemotherapy as a treatment for locally advanced accessory breast cancer [7–9]. Chemotherapy with anthracycline and taxane is generally recommended for the treatment of locally advanced accessory breast cancer, as for the usual form of breast cancer. One report on a locally advanced case indicated radical resection with reconstructive surgery for complete excision with a negative surgical margin [11]. Our patient underwent reconstructive surgery to fill the resulting skin defect with a local flap of the latissimus dorsi muscle to preserve shoulder joint movement. Reconstructive surgery must be recommended to relieve the negative effects of radical resection, including a large skin defect.

## 4. Conclusion

In this paper, we have described a very rare case of a male breast carcinoma arising in an accessory mammary gland. This case shows that neoadjuvant chemotherapy is an effective and tolerated therapy for advanced accessory breast cancer in the axilla, leading to curative resection combined with subsequent reconstructive surgery. We should take into account the possibility of accessory mammary carcinoma even in a male patient with adenocarcinoma in the axilla.
